# Whole-heart, free-breathing, three-dimensional myocardial BOLD MRI at 3T with simultaneous ^13^N-ammonia PET in canines

**DOI:** 10.1186/1532-429X-17-S1-Q126

**Published:** 2015-02-03

**Authors:** Hsin-Jung Yang, Damini Dey, Jane Sykes, John Butler, Avinash Kali, Ivan Cokic, Behzad Sharif, Debiao Li, Sotirios Tsaftaris, Piotr Slomka, Frank S Prato, Rohan Dharmakumar

**Affiliations:** 1 Cedars Sinai Medical Center, Los Angeles, CA, USA; 2 Lawson Health Research Institute, London, ON, Canada; 3 IMT Institute for Advanced Studies Lucca, Lucca, Italy

## Background

Myocardial BOLD MRI is an emerging non-contrast approach for the assessment of ischemic heart disease. However, current BOLD CMR methods (T2-weighted, T2*-weighted or bSSFP) are limited by poor spatial coverage and image artifacts (e.g. coil bias, B1 and B0 inhomogeneities). To address these limitations, we developed a fast, free breathing 3D T2 mapping technique at 3T that utilizes near 100% imaging efficiency. This quantitative BOLD approach, which can be performed within 5 minutes, permits full LV coverage during adenosine stress. In this study, we tested our approach in a canine model and validated our findings with simultaneously acquired ^13^N-ammonia PET perfusion data in a clinical PET-MR system.

## Methods

Mongrel dogs (n=3) were studied with a state of the art PET-MR system (Biograph mMR, Siemens Healthcare, Germany). After scouting and whole-heart shimming, fast 3D free-breathing T2 mapping sequence were prescribed at rest and under adenosine stress (140 mg/min/kg) with the following scan parameters: TR/TE =3.4/1.7 ms, flip angle = 15°, imaging resolution = 2 x 2 x 6 mm^3^ with 14 partitions, adiabatic T2 prep pulses (with T2 durations of 0, 24 and 55 ms) every other heart beat. Total acquisition time for whole LV coverage was 5 mins. For validation, dynamic ^13^N-ammonia PET scans were acquired along with MR data. PET images were analyzed using qPET (Cedars-Sinai Medical Center, US). Epi and endo-cardial contours were used to segment the myocardium and mean myocardial T2 (T2avg) values were measured from basal, mid and apical slices at rest and adenosine stress. The corresponding slices were matched to ^13^N-ammonia PET images and mean myocardial perfusion (Qavg) values were measured. Myocardial BOLD reserve (T2avg (stress):T2avg(rest)) and perfusion reserve (T2avg (stress):T2avg(rest)) were computed on a slice basis and regressed.

## Results

**Figure 1:**
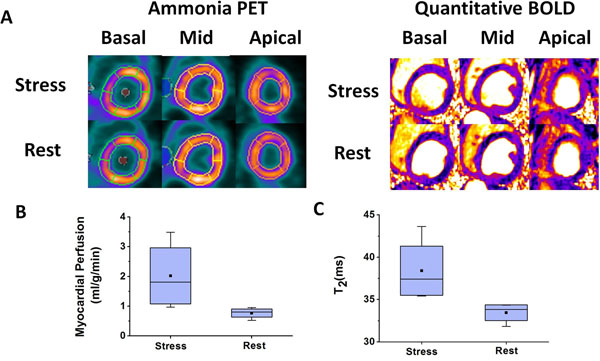
Representative basal, mid and apical short-axis images from BOLD and PET acquisitions at rest and stress are shown in (A). BOLD and PET images acquired at stress showed significant signal elevation compared to the corresponding rest images. Mean myocardial perfusion (Qavg) and BOLD (T2avg) measured from matched slices at rest and stress are shown in (B) and (C), respectively. Qavg during adenosine stress was significantly higher than at rest (B). Similar results were observed for BOLD (C).

A representative set of BOLD and PET images acquired from a canine under rest and adenosine stress are collected in Fig. 1. T2avg values measured under adenosine stress were significantly higher than at rest (T2avg: 33.5±1.0 ms (rest) vs. 38.4±3.1 ms (stress), p<0.05)). As expected, Qavg values were significantly higher during adenosine stress relative to rest (Qavg: 0.8±0.1 ml/mg/min (rest) vs 2.0±0.9 ml/mg/min (stress); p<0.05). Linear regression of BOLD and perfusion reserves showed high correlation (R^2^=0.67, p<0.05).

## Conclusions

The proposed fast 3D free breathing T2 mapping at 3T permits whole LV assessment of BOLD changes between rest and adenosine stress. The BOLD responses were very closely correlated with PET perfusion, suggesting that the proposed BOLD CMR method is a viable approach for imaging myocardial perfusion. Further studies are required to examine its utility in the setting of ischemic heart disease.

## Funding

This work was supported in part by National Heart, Lung, and Blood Institute HL091989.The content is solely the responsibility of the authors and does not necessarily represent the official views of the National Heart, Lung, And Blood Institute or the National Institutes of Health.

